# Analysis of Rutherford backscattering spectra with CNN-GRU mixture density network

**DOI:** 10.1038/s41598-024-67629-y

**Published:** 2024-07-23

**Authors:** Khoirul Faiq Muzakka, Sören Möller, Stefan Kesselheim, Jan Ebert, Alina Bazarova, Helene Hoffmann, Sebastian Starke, Martin Finsterbusch

**Affiliations:** 1 Institut für Energie- und Klimaforschung, Forschungszentrum Jülich GmbH, 52428 Jülich, Germany; 2 Jülich Supercomputing Centre, Forschungszentrum Jülich GmbH, 52428 Jülich, Germany; 3 Helmholtz-Zentrum Dresden-Rossendorf, 01328 Dresden, Germany

**Keywords:** Computational science, Characterization and analytical techniques

## Abstract

Ion Beam Analysis (IBA) utilizing MeV ion beams provides valuable insights into surface elemental composition across the entire periodic table. While ion beam measurements have advanced towards high throughput for mapping applications, data analysis has lagged behind due to the challenges posed by large volumes of data and multiple detectors providing diverse analytical information. Traditional physics-based fitting algorithms for these spectra can be time-consuming and prone to local minima traps, often taking days or weeks to complete. This study presents an approach employing a Mixture Density Network (MDN) to model the posterior distribution of Elemental Depth Profiles (EDP) from input spectra. Our MDN architecture includes an encoder module (EM), leveraging a Convolutional Neural Network-Gated Recurrent Unit (CNN-GRU), and a Mixture Density Head (MDH) employing a Multi-Layer Perceptron (MLP). Validation across three datasets with varying complexities demonstrates that for simple and intermediate cases, the MDN performs comparably to the conventional automatic fitting method (Autofit). However, for more complex datasets, Autofit still outperforms the MDN. Additionally, our integrated approach, combining MDN with the automatic fit method, significantly enhances accuracy while still reducing computational time, offering a promising avenue for improved analysis in IBA.

## Introduction

Ion Beam Analysis (IBA) comprises methods used to investigate the elemental and isotopic composition of samples by observing the nuclear and electronic interactions between MeV projectile ions (e.g., H, He, or I) and target electrons and nuclei^1,2^. IBA finds applications across diverse fields such as batteries, nuclear fusion, geology, space exploration, biology, and more. Each field poses unique questions, necessitating specific implementations of these methods tailored to the relevant elements, sampling volumes, and inquiries.

Different sub-methods are defined based on the investigated product, including Rutherford-backscattering spectrometry (RBS), nuclear reaction analysis (NRA), particle-induced X-ray emission spectroscopy (PIXE), among others. The energy loss of the projectiles and some resulting products in the sample encode depth information within the energy-resolved product spectra. In comparison to electron-based methods, ion-based techniques exhibit less statistical blurring (straggling) in the energy distributions concerning the energy loss. This results in appreciable depth resolutions ranging from a few micrometers down to less than 1 nanometer. When combined with the 2D surface scanning of the beam-spot, this provides 3D (tomographic) information capabilities.

Recent advancements in high-throughput and micro-beam end-stations^3^, alongside modern high-brightness accelerators and high-rate detectors and electronics, have substantially increased the volume of generated information over the past few decades. IBA spectra, especially charged particle spectra, encode depth and concentration information within the energy and intensity scales. The ion energy-loss induces a nonlinear coupling between signals related to different sample constituents. Each constituent may prompt various reactions with the projectile, resulting in contributions at different positions in the spectrum. These contributions possess individual heights (cross-sections) and depth information (product stopping) and may potentially overlap with other contributions.

Extracting sample information from these spectra has historically been time-consuming, relying on input conditions provided by IBA experts. Typically, fine-fitting using function minimizer like simplex methods leads to the final results. The speed of data evaluation and the necessity for manual interaction have emerged as significant limiting factors in the practical application of IBA. Although direct inverse calculations are possible^4^, they often yield results with large uncertainties due to numerical instabilities arising from uncertainties in experimental databases and experimental noise^5^. Additionally, deviations from Bragg’s law, straggling, and detector effects are usually not accounted for.

To address the limitations of traditional ion beam spectra evaluation, previous studies have employed deep learning-based methods, such as artificial neural networks (ANNs)^6–12^. The primary advantage of these methods lies in their significantly faster evaluation times, which, once appropriately trained, can assess thousands of spectra in less than a second, as opposed to days or weeks. Some earlier works^6,8–11^ explored the use of artificial neural networks (ANNs) in relatively simple sample scenarios, typically involving sample structures with fewer than three layers and fewer than five elements per layer. Exceptions include studies such as^7,12^, which assumed more than three layers. However, the number of elements per layer remained modest, with the total number of predicted depth profile parameters being fewer than 11. Given the success of these studies in relatively simple cases, there is significant interest in evaluating the accuracy of such methods in more complex situations. These scenarios may encompass not only increased layers and elements but also a greater number of depth profile parameters within the sample.

Rutherford Backscattering Spectrometry (RBS) analysis presents a classic example of an inverse problem, often entailing ambiguity due to the potential existence of multiple solutions for a given spectrum. This challenge is particularly pronounced in cases where overlaps occur in cross sections, further complicating the interpretation process. While the primary objective of RBS analysis is to derive a precise point estimate of elemental concentration, it is also crucial to articulate the associated uncertainty in these predictions. Traditional artificial neural networks (ANNs), as utilized in prior studies, do not inherently provide error estimation; therefore, additional steps must be taken to obtain predictions for the error estimates.

In this study, we depart from simple artificial neural network (ANN) regression and embrace Bayesian regression, enabling us to learn the conditional density (posterior) from the data. This transition provides insights into our understanding of the underlying distribution of parameters. Using the knowledge of the posterior not only facilitates the calculation of the best estimate for the Elemental Depth Profile (EDP) given an input spectrum but also allows for the assessment of their uncertainty, identification of correlations, and even discern multiple EDP solutions that reproduce the input spectrum.

Several approaches exist to learn a conditional density from a set of observations, including normalizing flow^13^, conditional variational auto-encoders^14^, Generative Adversarial Network^15^, and likelihood-free approaches as in^16^. However, in this study, we opt for a mixture density network (MDN)^17^.

MDNs offer advantages in terms of interpretability, cost-effective evaluation, and efficient sampling of resulting posteriors. Moreover, MDNs provide an analytical formula for calculating the expectation value and covariance of predictions, alleviating the need for laborious sampling procedures to evaluate them from the posterior density. This feature thus enhances computational efficiency. Furthermore, our numerical experiments, outlined in Supplementary Material A, demonstrate that MDNs yield more accurate point estimates compared to neural network-based point-regression approaches like ANNs. This advantage likely stems from MDNs’ ability to capture the distribution of outcomes, enabling them to handle data points that deviate from the main trend or exhibit unusual behavior more effectively.

## RBS simulation and automatic fit

In the introduction, Rutherford Backscattering Spectrometry (RBS) was introduced as an ion beam analysis (IBA) technique that utilizes the spectrum of back-scattered particles to estimate the elemental depth profile of a sample. In this section, we provide a brief overview of the theoretical foundations of RBS and describe the typical evaluation process of an RBS spectrum.

**Figure 1:**
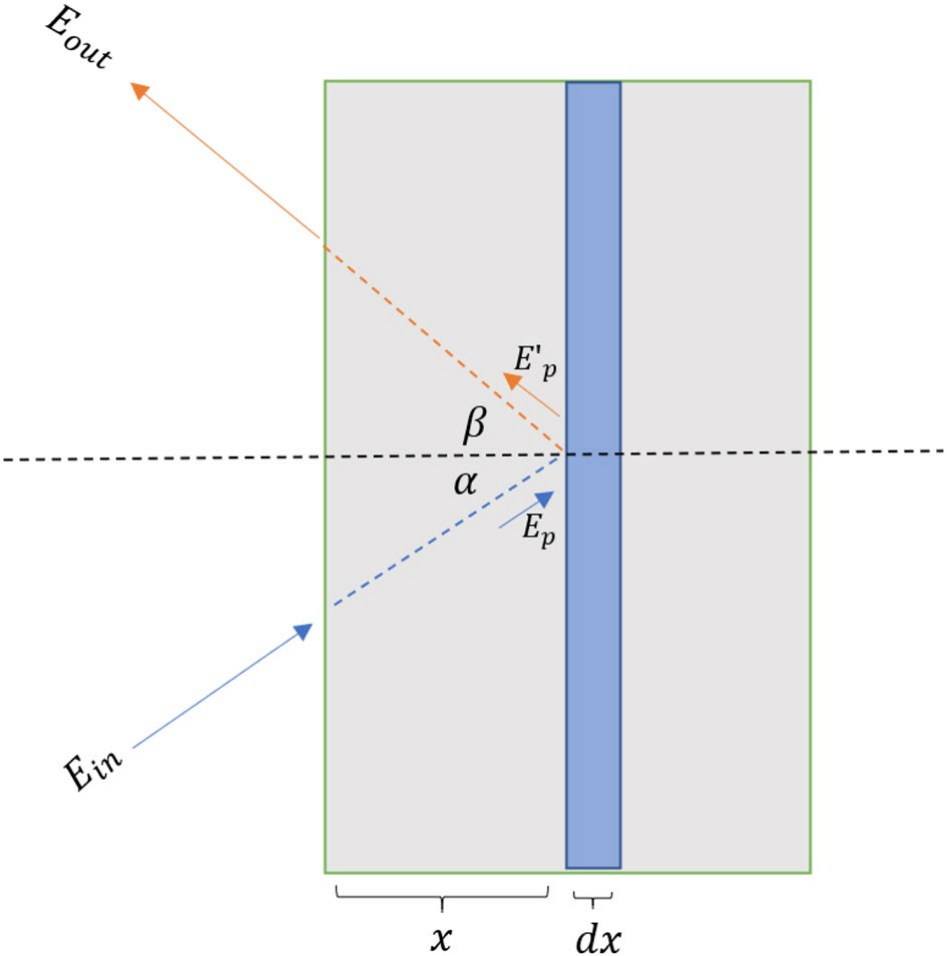
Schematic view of an ion beam coming into a sample and producing an ion with a detected energy $E_{out}$.

**Figure 2:**
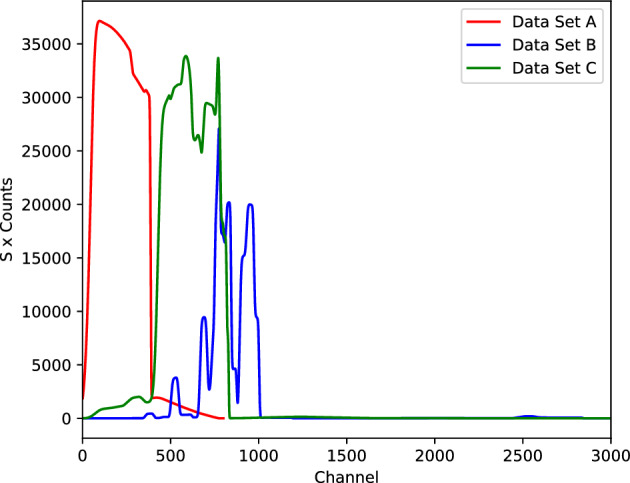
SIMNRA predictions of the RBS spectrum of projectile ions from three sample setups, as discussed in section “Methods”. Here, the scale factors *S* for data sets A, B, and C are $S\in \{0.05, 10, 1\}$, respectively.

Figure 1 illustrates a schematic representation of an ion beam (*p*) with energy $E_{in}$, directed at a sample with an incident angle $\alpha$ normal to its surface. One of the resulting products of the bombardment is the same ion (*p*) exiting the sample at an angle $\beta$ with a detected energy $E_{out}$. For simplicity, we assume the sample is homogeneous, implying a constant elemental concentration at all depths. An inhomogeneous sample can be modeled by a stack of homogeneous ones, thus it is just a straightforward generalization of our discussion here.

Considering the homogeneous sample as a series of infinitesimally thin layers, we can predict the number of back-scattered ions (*p*) as a function of the detected energy $E_{out}$ by summing the contributions from each individual layer. To calculate the contribution from a layer with thickness *dx* (refer to Fig. 1), containing $N_i$ atoms of type $A_i$, the number $N_{p}$ of back-scattered particles is related to the layer thickness *dx* as

1 $$\begin{aligned} \frac{dN_p}{N_p} = dx \,d\Omega \sum _i \rho _i(x) \frac{d\sigma _i (E_p)}{d\Omega } \end{aligned}$$

Here, $\rho _i(x)$ is the number density of atom species $A_i$, $\Omega$ is the detector solid angle, and $E_p$ is the energy of the projectile before scattering (see Fig. 1). In laboratory, the number of back-scattered particle $N_p$ is measured as a function of the detected energy $E_{out}$. Therefore, to connect with the measured quantity, we must express the depth *x* in terms of $E_{out}$. The strategy is to relate *x* with the energy $E_p$ of the ion before scattering. The relation is given by the stopping power equation

2 $$\begin{aligned} \frac{dE_p}{dx} = -\rho (x) S(E_p, \rho _i(y)) \end{aligned}$$

Here, $\rho (x)=\sum _i \rho _i(x)$ is the total number density and $S(E, n_i(y))$ is the stopping power. After the scattering at depth *x*, the back-scattered energy $E'_p$ is related to $E_p$ as

3 $$\begin{aligned} E'_p = k E_p \end{aligned}$$

where *k* is a kinematical factor which depend on the reaction type, the released energy of the reaction (*Q*), the masses of all particle involved in the reaction, and the scattering angle $\theta =\alpha +\beta$. Using Eqs. (1, 2, 3), the calculation of the number of back-scattered particles as a function of the detected energy $E_{out}$ bin can be performed. While the underlying physics of simulating ion backscattering is relatively straightforward, it’s important to note that modern simulation softwares(for review and comparison, see^5,18^), such as SIMNRA^19^, accounts for more nuanced effects. These include straggling (the broadening of the ion’s energy distribution), sample characteristics like roughness and porosity, and detector-specific effects such as pile-up and finite resolution.

Figure 2 illustrates RBS spectra of projectile ions, simulated using SIMNRA, derived from three sample setups (data set A, B, and C from section “Methods”) that will be investigated in this work. The horizontal axis denotes the channel number, which is linearly associated with detected energy following calibration of the experimental setup. Meanwhile, the vertical axis represents the count of backscattered ions corresponding to the energy within each channel. Given the nature of the measurement, which involves counting, the count within each channel conforms to Poissonian statistics.

RBS analysis entails estimating the elemental depth profile (EDP) of a sample based on a measured RBS spectrum. The EDP is characterized by the EDP parameters, which represent the thickness for each element in each sublayer structure of the sample. Given that we solely possess a simulator for the forward process, this analysis constitutes an instance of an inverse problem. Typically, the analysis involves iteratively comparing the forward predictions from SIMNRA to the measured spectrum until a convergence criterion is satisfied. This method of estimating EDPs will be referred to as automatic fitting or Autofit.

The general procedure of an Autofit typically involves minimizing a $\chi ^2$ function, which quantifies the dissimilarity between the input spectrum and the simulated one given a set of EDP parameters. Ideally, a global minimizer such differential evolution or evolutionary annealing (see^20^ for performance comparison in the RBS analysis case), should be used to obtain the global minimum. Such an algorithm also has the advantage of not requiring starting EDP parameters to initiate the procedure. However, it generally comes with a higher computational cost compared to a local minimizer, such as adaptive Nelder–Mead^21^. Given that our aim is to perform massive analysis of thousands of spectra within limited time constraints, a good and efficient optimizer is therefore preferred.

In this study, Autofits are conducted using the AutoNRA program, a custom Python application developed to augment the capabilities of the SIMNRA program. AutoNRA features a graphical user interface built with Qt PySide and supports running multiple SIMNRA fits simultaneously using multi-threading or multi-processing. These features enable us to utilize nearly 100% of the available computing resources. For our specific use case, Autofits are typically executed using 120 threads on a Windows PC equipped with an AMD Ryzen Threadripper CPU (64 cores).

In our implementation, the Autofit fit minimizes the following $\chi ^2$ function :

4 $$\begin{aligned} \chi ^2({\textbf {y}}) = \sum _i^{N} \left( \frac{x_i-\text {SIMNRA}_i({\textbf {y}})}{\sqrt{x_i+1}}\right) ^2 \end{aligned}$$

Here, *N* is the spectrum length, $x_i$ represents the spectrum value for the channel *i*, and SIMNRA$_i$(${\textbf {y}}$) signifies the prediction by the SIMNRA program given the EDP parameters ${\textbf {y}}$. As mentioned before, the spectrum data is inherently Poissonian, the uncertainty in the spectrum is assumed to be the square root of itself plus a unit shift factor to avoid numerical issue when $x_i=0$. Minimization of $\chi ^2$ is carried out using adaptive Nelder–Mead^21^. One of key parameters in the Nelder–Mead algorithm, as implemented in the Python SciPy package, is *Maxfev*. This parameter determines the maximum number of function calls-such as the $\chi ^2$ function in our case-before the algorithm terminates. In our study, we set *Maxfev* to be $200 \times D$, where *D* represents the number of EDP parameters. Additionally, the Nelder–Mead algorithm is restarted every 500 function calls using the minimum value obtained from the previous run as the new starting point.This approach helps the algorithm find a better minimum in our experience.

To evaluate the accuracy of predicted EDP parameters represented by ${\textbf {y}}$ (in the Autofit method, this is simply the minimum of $\chi ^2({\textbf {y}})$), we can perform a $\chi ^2$-test to check if its corresponding SIMNRA spectrum agrees with the input spectral data *x*. Assuming that the spectral data is normally distributed with variance $x +1$ around the SIMNRA prediction, the resulting $\chi ^2({\textbf {y}})$ follows a $\chi ^2$-distribution with *N* degrees of freedom, denoted as $\chi ^2_{N}$, where *N* is the length of the spectrum. We note that the spectral data is Poissonian by nature with a standard deviation of $\sqrt{x}$. Therefore, the assumption above relies on the similarity between Gaussian and Poissonian distributions. This typically happens at large *x*, which also implies $\sqrt{x_i+1} \approx \sqrt{x}$.

Given the statement above, then the $\chi ^2_{N}$ distribution can act as an ideal distribution against which we can compare the observed $\chi ^2({\textbf {y}})$. This comparison allows us to assess whether the EDP parameters ${\textbf {y}}$ remain a viable solution to the inverse problem: given *x*, find ${\textbf {y}}$. In a strict statistical hypothesis testing method, one can choose a desired level of significance $\alpha$, for example $\alpha = 0.05$. Then a solution ${\textbf {y}}$ is rejected if $\chi ^2({\textbf {y}})$ is larger than the $100(1-\alpha )$ percentile of the ideal distribution, otherwise it is accepted. It is worth mentioning that the actual value of $\chi ^2$ can be much lower than the ideal $\chi ^2_{N}$. This discrepancy occurs when many channels have $x_i$ values close to 0. In such cases, the contributions from these channels to the $\chi ^2$ value can be negligible, resulting in a smaller $\chi ^2$ value than expected.

It is important to mention that when using experimentally measured spectra, the statistical test as described above, which rely on strict assumptions, may lose its validity. This is mainly due to SIMNRA’s dependency on input data derived from experimental measurements, including cross-sections and stopping power, which inherently possess limited accuracy. Consequently, assessments based on such data may incorrectly label fits with large $\chi ^2$ values as poor, even when they are indeed satisfactory. In this study, our analyses solely use simulated spectra generated by SIMNRA. Consequently, the aforementioned issue does not apply to our case. Therefore, we can confidently utilize the above statistical test to assess the quality of our evaluation.

## Methods

### Density estimation using mixture of Gaussian

The goal of this work to estimate the posterior, or the conditional density $p({\textbf {y}}|x)$ of minmax-scaled EDP parameters ${\textbf {y}} = (y_1, y_2,\ldots , y_D)$, given a standard-scaled spectrum *x*. In the mixture density framework, we assume that the posterior can be approximated by a mixture of Gaussian

5 $$\begin{aligned} p({\textbf {y}}|x)&\approx \sum _{i=0}^M \pi _i (x) \mathscr {N}_i( {\textbf {y}}|x) \end{aligned}$$

6 $$\begin{aligned} \mathscr {N}_i({\textbf {y}}|x)&= \frac{1}{{(2\pi )}^{D/2}}\frac{1}{\prod _j^D\sigma _j^i (x)} \exp \left( -\frac{1}{2}\sum _{j=0}^D\left( \frac{y_j-\mu _j^i(x)}{\sigma _j^i(x)}\right) ^2\right) \end{aligned}$$

Here *M* is the number of Gaussian components, *D* is the number of EDP parameters, while $\pi _i$, $\mu _j^i$ and $\sigma _j^i$ are the mixture fraction of the *i*-th Gaussian component, the center of the *i*-th Gaussian component that correspond to the *j*-th EDP parameter, and its standard deviation respectively. These quantities are the outputs of our model and are functions of *x*. Equation (5) can be rewritten as

7 $$\begin{aligned} p({\textbf {y}}|x)&= \frac{1}{{(2\pi )}^{D/2}}\sum _i^M \exp \left( -\frac{\phi _i(x, {\textbf {y}})}{2}\right) \end{aligned}$$

8 $$\begin{aligned} \phi _i(x, {\textbf {y}})&= \sum _{j=0}^D\left( \frac{y_j-\mu _j^i(x)}{\sigma _j^i(x)}\right) ^2 -2 \log \left( \frac{\pi _i(x)}{\prod _j^D\sigma _j^i (x)}\right) \end{aligned}$$

Given the posterior, the expectation value and variance of the predictions for ${\textbf {y}}$ can be calculated as (the input variable *x* is being suppressed for conciseness reason)

9 $$\begin{aligned} E\left[ {\textbf {y}}|x\right]&= \int {\textbf {y}} \sum _i \pi _i \mathscr {N}_i d^D{\textbf {y}} = \sum _i \pi _i \varvec{\mu }^i\end{aligned}$$

10 $$\begin{aligned} \text {Cov}_{ij}\left[ {\textbf {y}}|x \right]&= \int \left( y_i-\sum _k \pi _k {\mu }^k_i\right) \left( y_j-\sum _k \pi _k {\mu }^k_j\right) \sum _l \pi _l \mathscr {N}_l \,d^D{\textbf {y}}\nonumber \\ {}&= \sum _k \pi _k ({\sigma _i^k})^2 \delta _{ij} + \sum _k \pi _k \mu _i^k \mu _j^k - \sum _{k,l} \pi _k \pi _l \mu _i^k \mu _j^l \end{aligned}$$

where $\delta _{ij}$ is the Kronecker delta and we have used the following identities :

11 $$\begin{aligned} \int y_i \,\,\mathscr {N}_l d^D{\textbf {y}}&= \mu _i^l\end{aligned}$$

12 $$\begin{aligned} \int y_i y_j \,\,\mathscr {N}_l d^D{\textbf {y}}&= \delta _{ij} {\sigma _i^l}^2 + \mu _i^l \mu _j^l \end{aligned}$$

Note that even though the covariance of the individual Gaussian components is diagonal, the resulting Gaussian mixture has non zero off-diagonal covariance. Using the fact that $\sum _i \pi _i =1$, we can also rewrite (10) as

$$\begin{aligned} \text {Cov}_{ij}\left[ {\textbf {y}} |x\right]&= \sum _k \pi _k ({\sigma _i^k})^2 \delta _{ij} + \sum _k \pi _k \mu _i^k \mu _j^k - 2\sum _{k,l} \pi _k \pi _l \mu _i^k \mu _j^l + \sum _{k,l, m}\pi _m \pi _k \pi _l \mu _i^k \mu _j^l\\&= \sum _k \pi _k \left[ ({\sigma _i^k})^2 \delta _{ij} + \left( \mu _i^k-\overline{\mu }_i\right) \left( \mu _j^k-\overline{\mu }_j\right) \right] \end{aligned}$$

where

13 $$\begin{aligned} \overline{\mu }_i&= \sum _l \pi _l \mu _i^l = E[y_i] \end{aligned}$$

Given the covariance $\text {Cov}_{ij}[{\textbf {y}}]$, the variance is identified as the diagonal part of the covariance^22^:

14 $$\begin{aligned} Var[y_i] = \sum _k \pi _k \left( ({\sigma _i^k})^2 + \left( \mu _i^k-\overline{\mu }_i\right) ^2 \right) \end{aligned}$$

While our model inherently provides a posterior distribution given an input spectrum, the computational burden of handling the full density can become prohibitive, especially when thousands of spectra need evaluation. This is where opting for a point estimate is advantageous. In this case, the information on the full posterior is summarized in the form of uncertainty estimates. Typically, the point estimate of interest is derived from the global maximum of the posterior distribution, known as the Maximum a Posteriori or MAP estimate. However, the challenge lies in the fact that a mixture of *M* Gaussian distributions can contain anywhere from one to *M* local minima, rendering the task of finding the global maximum of the posterior computationally expensive.

In this work, to predict a point estimate and quantify its uncertainty, we use the mean and standard deviation of of the predicted posterior distribution. Thus, the point estimate is given by (9), while its uncertainty is given by (14).

### MDN architecture and training

**Figure 3:**
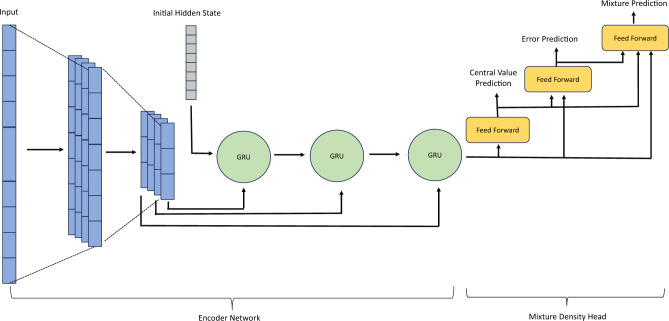
Schematic of the CNN-RNN Model used in this work.

The Mixture Density Network (MDN)^17^ utilized in this study comprises two integral components: the Encoder Network (EN) and the Mixture Density Head (MDH). The EN encodes the spectrum into a vector, serving as input to the MDH, which produces the mixture coefficient $\pi _i$, the center of the Gaussian component $\mu ^{(i)}$, and its standard deviation $\sigma ^{(i)}$ as output.

The EN, responsible for transforming the input spectrum into a format digestible by the MDH, is implemented using a convolutional neural network (CNN). Given that a spectrum can be viewed as a 1D image, CNN is a natural choice, applying learnable convolutional filters to automatically extract features. In previous spectroscopic analyses^23–25^, CNN has demonstrated its prowess in capturing intricate details inherent in spectroscopic data.

In CNN, reduction operations like pooling and non-unit stride reduce data dimensionality, resulting in a per-channel output from the CNN that is also spatially reduced. Despite this reduction, the numerous output channels often lead to a high-dimensional output after flattening. To address this, a gated recurrent unit (GRU)^26^ is employed to sequentially process these input channels, effectively reducing the output dimension. Our experimentation has validated that an MDN featuring a combined CNN+GRU architecture as the EN consistently outperforms its counterpart with a sole CNN EN, particularly in the IBA case (see our ablation study in Supplementary Material A).

The GRU, a type of recurrent neural network (RNN), is specifically designed to capture long-term dependencies within sequential data. Serving as a computationally efficient alternative to the long short-term memory (LSTM) network^27^, the GRU incorporates a sophisticated gating mechanism involving a reset gate for determining the amount of previous state information to forget and an update gate for deciding how much previous state information to pass to the next time step. In our approach, the multi-channel output from the CNN is sequentially processed by the GRU to generate a final representation of the input data before being fed into the MDH unit.

The architecture of the CNN-GRU-based MDN is depicted schematically in Fig. 3. During the forward step, a preprocessed spectrum *x* is inputted into a 1D CNN, which outputs several channels of spatially reduced sub-spectra. These sub-spectra are subsequently processed in sequence by the GRU network, whose hidden states are initialized with zeros. The final output (hidden states) of the GRU network serves as a summary statistic *h*, which is then fed into the MDH. In the MDH, a simple feed-forward network (without hidden layers) transforms the summary statistic *h* into predictions for $\mu$. To predict $\sigma$, the output $\mu$ is first concatenated with *h*. Then, a multilayer feed-forward network, with a softplus function as the output layer activation, transforms the input into the prediction of $\sigma$. Finally, $\mu$ and $\sigma$ are concatenated with *h*, and a feed-forward network (without hidden layers) transforms the concatenated inputs into predictions for the mixture coefficient $\pi$.

The MDN training process involves minimizing negative log posterior loss, with additional steps taken to prevent numerical instability (see Supplementary Material B for details). Throughout training, we employ the AdamW optimizer^28^ to minimize the loss function. For this study, we set the total number of training epochs to 100, divided into 5 steps. Within each step, we utilize different learning rates and implement early stopping if there is no improvement in the loss function of the validation data for more than 20 epochs. Additionally, in each step, the network initializes with parameters that yielded the best validation loss in the preceding step. The entire model and training process are implemented using the PyTorch package. On a PC equipped with an NVIDIA GPU RTX A-4000, the training procedure typically completes in less than 5 min when using 10,000 training data samples.

### Data generation and the model

Adopting a data-driven approach, the machine learning methodology utilized in this study heavily depends on a significant amount of training data. This data is acquired by first establishing a range of Elemental Depth Profile (EDP) parameters and then sampling them under the assumption of a uniform distribution.

Once the range of these parameters is established, a software program capable of performing forward calculations, such as SIMNRA^19^, is employed to compute the corresponding spectra. Subsequently, these spectra are rounded to the nearest integer and are subjected to the application of Poisson noise. This step is essential for emulating experimentally produced spectra and serves as a data augmentation technique to improve the performance of a machine learning model^29^.

In order to assess the learning capacity of our models with respect to the inverse map as the complexity (defined in terms of the number of EDP parameters) of the problem escalates, we generate three types of training data:

1. **dataset A:** This dataset corresponds to samples containing two elements, Deuterium (D) and Tungsten (W), arranged in two layers, each with a constant concentration of D and W. The Elemental Depth Profile (EDP) for this dataset is defined by four parameters, indicating the thickness of each element in the respective layers. The dataset *A* represents an easy task with no ambiguity for the inverse map. Consequently, we choose MDN model with a single component ($M=1$) to learn the posterior distribution for this case. We have verified that using $M>1$ does not improve the accuracy of our model’s point estimates, see Supplementary Material A for details. The MDN model for this dataset consists of a 1D CNN with four convolutional layers, two layers of bi-directional GRU network with 128 hidden states, and a Mixture Density Head (MDH). In the convolutional part, we utilize 45, 35, 30, and 25 kernels for the first, second, third, and last layer, respectively, with kernel sizes 7, 5, 5, and 3, along with leaky-ReLU non-linearity with the negative-slope parameter 0.01 after each layer. For the MDH, as detailed in the previous section, we employ a feed-forward network without a hidden layer for the Gaussian center ($\mu$) and mixture ($\pi$) predictions. Regarding the $\sigma$ prediction, we use a feed-forward network with hidden layer sizes (300, ).
2. **dataset B:** This dataset assumes that the sample is composed of three distinct layers, each containing lithium (Li), carbon (C), oxygen (O), fluoride (F), and nickel (Ni). In total, there are 15 parameters that specify the thickness of each element within each layer. Notably, the number of Elemental Depth Profile (EDP) parameters in this dataset exceeds that of dataset A, signifying a higher level of complexity in this particular sample. To model the posterior distribution of the mapping $x\rightarrow y$, our Mixture Density Network (MDN) employs 8 Gaussian components ($M=8$). In the Encoder Network (EN) segment, a Convolutional Neural Network (CNN) is utilized with 6 convolutional layers, featuring 55, 50, 45, 35, 30, and 25 channels, with kernel sizes of 15, 12, 10, 7, 5, and 3 for each respective layer. The stride alternates between 1 and 2, and no pooling operation is applied. The Gated Recurrent Unit (GRU) networks in the EN consist of 2 bidirectional GRU layers, each with 128 hidden states. In the Mixture Density Head (MDH) unit, a feed-forward network without a hidden layer is employed for the Gaussian center ($\mu$). For the predictions of $\pi$ and $\sigma$, feed-forward networks with hidden layer size of ( 100, ) and (800, ) are used, respectively.
3. **dataset C:** This dataset involves samples comprising five layers, each containing Lithium (Li), Carbon (C), Oxygen (O), Zirconium (Zr), Lanthanum (La), and Tantalum (Ta). In the first layer, aside from the mentioned elements, there are also Hydrogen (H), Carbon (C), and Cobalt (Co). Furthermore, in the last layer, Carbon (C) is absent. This dataset represents a scenario with a large number of EDP parameters (30 parameters) and a high degree of cross-section overlap. The MDN for this dataset assumes the same architecture as that used in dataset B. The difference is that now 10 Gaussian components ($M=10$) are used instead of 8.

The three datasets, in addition to having an increase in the number of EDP parameters, also exhibit an increase in the length of the region of interest (RoI). A region of interest (RoI) refers to specific segments of the RBS spectrum that are particularly relevant for extracting meaningful compositional information about the sample. In our case, the first 100 channels are excluded from the RoI due to their low information content. Furthermore, regions where counts are always zero under the variation of the EDP parameters are also excluded from the ROI. With this definition in mind, the RoI for data set A is determined to be between channels 100 and 800, resulting in an RoI length of 700. For data set B, the RoI spans from channels 320 to 1200 and 2420 to 2850, with a total RoI length of 1310. For data set C, the RoI ranges from channels 100 to 2300, giving an RoI length of 2200.

In Fig. 2, we present an RBS spectrum from a randomly chosen sample from data sets A, B, and C. We can observe that these three data sets also represent cases with increasing levels of ambiguity. For data set A, there is no backscattering from deuterium atoms in the sample, resulting in their absence in the spectra and thus no overlap occurs. Comparing the spectra from data set B (blue line) and data set C (green line), we clearly see that the peaks in the spectrum from data set B are more separated than those in data set C. In the spectrum from data set C, the region with channel numbers less than 850 shows the highest level of peak overlap, particularly from oxygen (O), tantalum (Ta), cobalt (Co), lanthanum (La), and zirconium (Zr).

Given the sample setups for the three data sets, we generate 100,000 instances for training. Additionally, we create 2000 test and 1000 validation data points to evaluate the performance of the trained model. Across all these datasets, the values of EDP parameters are uniformly distributed within the interval [50, 5000] atoms/cm$^2$.

’

### Data preprocessing

Before being input into the training module, the training data undergoes dimensional reduction through several steps for improved model simplicity and faster training times. This reduction is implemented in the following manner:

1. Bin-Merging: Initially, bin-merging is performed, where four bins are merged into a single bin. Given a spectrum length *N* and the length of the merged bins $N_{\text {merge}}$, the final spectrum length after merging becomes ${\big \lceil N/N_{\text {merge}}\big \rceil }$.
2. Dimension Reduction based on Variance: Following bin-merging, portions of the spatially reduced spectrum with low variance counts are discarded. This step involves removing bins with counts that exhibit low variability across all training data. Specifically, a channel is identified as having low-variance counts if, across all training data, the standard deviation of the counts in that channel lies within the 95% percentile (3 $\sigma$) of Poisson fluctuations of the mean data value. Thus, given the *i*-th bin, we remove this bin if
  15 $$\begin{aligned} std(C_i)=\sqrt{\frac{1}{N_{data}}\sum _k \left( C_{i}^{k} - \bar{C}_i\right) ^2 }\le 3 \sqrt{\bar{C}_i} \end{aligned}$$
  In this equation, $N_{\text {data}}$ represents the number of data points, $C_i^k$ is the count in the *i*-th bin for the *k*-th spectrum, and $\bar{C}i= \left( \sum _k C_i^k\right) /N{\text {data}}$ is the mean count. Essentially, this method determines that if the count data in a bin *i* satisfy (15), then all the data in that bin are considered as random fluctuations from the same data, and consequently, the bin discarded. It’s important to note that this technique shares similarities with Principle Component Analysis (PCA). The primary distinction lies in the fact that PCA involves iterative rotations of the data (within the channel space) to maximize variance along newly rotated axes. In contrast, our approach refrains from using PCA to maintain the interpretability of the input channels.
3. Smoothing of Dimensionally Reduced Spectrum: Smoothing is a common preprocessing technique and used to remove noise and enhance the signal in spectral analysis^30^. To smooth the dimensionally reduced spectrum, noise suppression is applied using Savitzky–Golay filtering^31^.
4. Scaling: Finally, the spectrum and the target Elemental Depth Profiles (EDPs) are scaled using standard and min-max scaling respectively. Here, Standard scaling entails adjusting the spectrum based on its standard deviation, ensuring consistency and normalization across the data range. The min-max scaling rescale the EDPs to fit within the interval [0,1].

This series of steps in dimensional reduction and preprocessing aims to streamline the data and optimize it for more efficient and effective training of the machine learning models.

## Results and discussion

In accordance with the methodology described in the preceding section, we executed the machine learning pipeline and conducted a comparison with automatic fit methods to evaluate the test data. All computations were performed on a PC running Windows 11, equipped with 128 GB of RAM, an AMD Ryzen Threadripper PRO 5995WX CPU with 64 cores, and an Nvidia RTX A-4000 GPU. Specifically, the generation of training data and automatic fits occurred on the CPU, while both training and inference steps were executed on the GPU. Table 1 displays the run times for both the machine learning pipeline and the inference process.

**Table 1:** The runtime (in hours) for the machine learning pipeline, tasked with predicting *D* EDP parameters, is reported for datasets A, B, and C. The pipeline encompasses the generation of 103,000 spectra, a training process with 50,000 spectra, and testing and benchmarking using 2000 test data points.

Sample	*D*	Data generation (103k)	Training (50k)	Autofits (2k)	ML + Autofits (2k)
Dataset A	4	0.1	0.5	1.5	1
Dataset B	15	0.5	0.75	15	5
Dataset C	30	0.5	0.75	30	9

To systematically analyze the outcomes of the numerical experiment, we start by evaluating the accuracy of our model’s predictions. Subsequently, we present a comparative analysis between the results of the SIMNRA simulation using inputs from our model (back-simulation) and the outcomes of our goodness-of-fit test. We then discuss of the uncertainty predictions generated by our model. Finally, we explore a combined approach that proves superior to both machine learning (ML) and automatic fit methods.

### Accuracy of the model predictions

The accuracy of the point estimate of EDP parameters, given by the expectation value $\langle {\textbf {y}} \rangle$ calculated using a predicted posterior distribution, can be evaluated by comparing the prediction to the corresponding target value. To quantify this, we use the absolute relative error:

16 $$\begin{aligned} \varepsilon = \left| \frac{prediction-target}{target}\right| \end{aligned}$$

**Figure 4:**
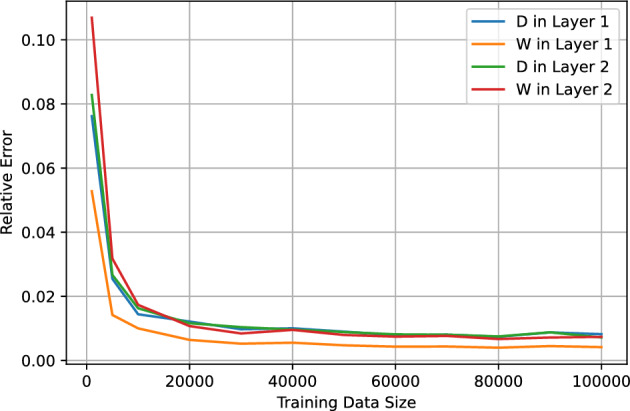
The median of relative error of the test data from dataset A as a function of the training data size.

**Figure 5:**
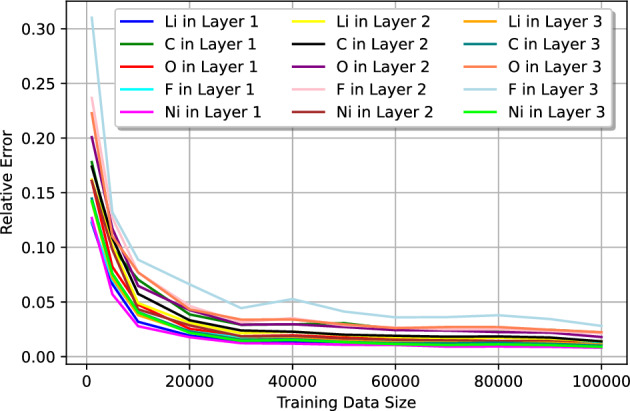
The median of relative error of the test data from dataset B as a function of the training data size.

In Figs. 4 and 5, we present the median relative error (MRE) computed for 2000 test instances derived from datasets A and B, respectively, plotted against varying sizes of training data. Notably, in dataset A, we observe a remarkable convergence of the MREs to $\lesssim 2\%$ levels with a training size of 10,000. Beyond this point, the MREs stabilizes at approximately 0.8% as the training data size increases further. In contrast, the analysis of dataset B reveals a discernible elevation in the MRE compared to that observed in dataset A. This discrepancy is attributed to the intrinsic complexity of dataset B and the higher number of parameters being predicted. Nonetheless, even with a modest training size of 30,000, we achieve MRE values below 5% across all EDP parameters. Moreover, with a training set size of 50,000 or greater, the MREs can be further refined to $\lesssim 3\%$.

**Figure 6:**
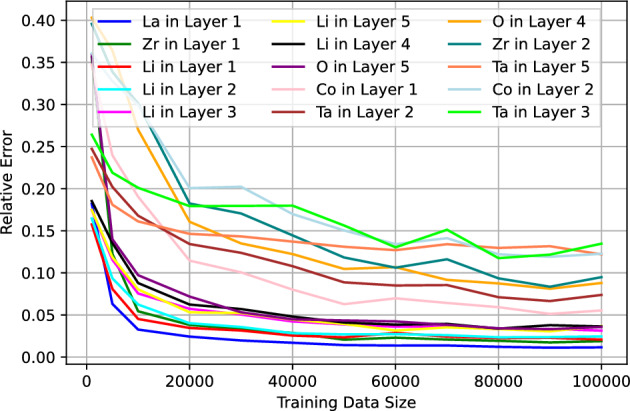
The median of relative error of the test data from dataset C as a function of the training data size for the 15 best predicted parameters.

**Figure 7:**
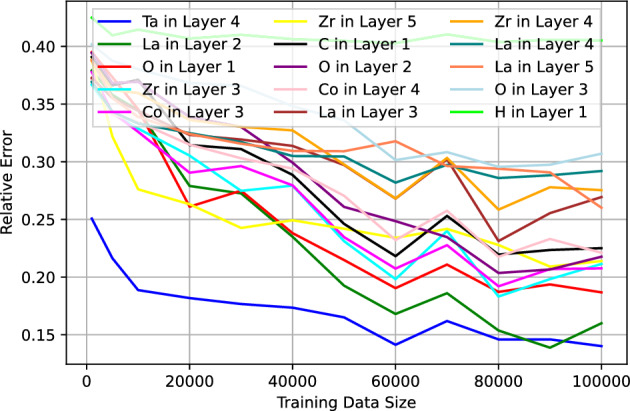
The median of relative error of the test data from dataset C as a function of the training data size for the 15 worst predicted parameters.

Turning our attention to dataset C, which presents the most complex scenario, we examine Figs. 6 and 7. These figures illustrate the MRE plotted against the training size for both the 15 best and worst predicted Elemental Depth Profile (EDP) parameters. Upon closer inspection, it becomes evident that only a select few EDP parameters, particularly those corresponding to layer 1, demonstrate MRE values of $\lesssim 5\%$ at the training size 50,000 or larger. Additionally, parameters pertaining to shallower layers seems to exhibit higher prediction accuracy compared to those from deeper layers. This observed trend can be attributed to the diminishing energy of back-scattered particles as we traverse deeper into the material. Consequently, the signals become increasingly overlapped. This ambiguity ultimately leads to diminished accuracy in predicting parameters associated with deeper layers.

In summary, our model performs exceptionally well in terms of point estimates for datasets A and B. However, for dataset C, while it accurately predicts some EDP parameters, the accuracy exceeds 5% for the remaining parameters, even with a large number of training samples.

### Autofits and goodness-of-fit test

Due to the ambiguous nature of the inverse mapping, sometimes two different sets of EDPs correspond to same input spectra. Thus, examining ground-truth relative error alone can be misleading. To assess the quality of the predicted EDPs, we therefore employ an additional metric, which is the $\chi ^2$ of the resulting simulated spectrum, see (4) and the discussion in section “RBS simulation and automatic fit”.

In Table 2, we present the statistical profile of the observed $\chi ^2$ values for the 2000 test data, obtained from our model trained with 10,000 (MDN10k), 50,000 (MDN50k), 100,000 (MDN100k) training samples. Additionally, we also include the profile of the $\chi ^2$ distribution with $\nu =700$ degrees of freedom, corresponding to the ideal case where the predicted EDPs coincide with the true EDPs used to generate the data (see discussion in section “RBS simulation and automatic fit”). Note that the number of degrees of freedom always coincides with the RoI length, which is 700 for data set A. For comparison, we also show the $\chi ^2$ values from the Autofit method.

**Table 2:** Statistical profile (percentiles, mean and standard deviation) of $\chi ^2$ of the test data of dataset A, where the predicted EDPs are from the Autofit and our MDN trained with 10,000, 50,000, and 100,000 training data. Profile from an ideal $\chi ^2$ distribution with $\nu$ degrees of freedom is also shown.

Method	Percentiles							Mean	Std
	5th	10th	25th	50th	75th	90th	95th		
MDN10k	667	686	725	801	1066	2722	3972	1308	1720
MDN50k	639	654	680	720	786	1213	2422	949	1058
MDN100k	636	651	677	712	765	995	1660	868	639
Autofit	626	640	667	707	1360	6083	15,380	5640	28,309
$\chi ^2_{\nu =700}$	640	652	674	699	725	748	763	700	37

**Table 3:** Statistical profile (percentiles, mean and standard deviation) of $\chi ^2$ of the test data of dataset B, where the predicted EDPs are from the automatic fit and our MDN trained with 10,000, 50,000, and 100,000 training data. Profile from an ideal $\chi ^2$ distribution with $\nu$ degrees of freedom is also shown.

Method	Percentiles							Mean	Std
	5th	10th	25th	50th	75th	90th	95th		
MDN10k	1553	1671	1967	2486	3626	5214	7233	3250	2585
MDN50k	1097	1140	1214	1302	1391	1523	1651	1343	536
MDN100k	1091	1131	1208	1293	1389	1509	1630	1325	236
Autofit	1004	1048	1114	1199	1283	1358	1478	19,735	109,886
$\chi ^2_{\nu =1310}$	1227	1245	1275	1309	1344	1376	1395	1310	51

**Table 4:** Statistical profile (percentiles, mean and standard deviation) of $\chi ^2$ of the test data of dataset C, where the predicted EDPs are from the automatic fit and our MDN trained with 10,000, 50,000, and 100,000 training data. Profile from an ideal $\chi ^2$ distribution with $\nu$ degrees of freedom is also shown.

Method	Percentiles							Mean	Std
	5th	10th	25th	50th	75th	90th	95th		
MDN10k	1.71e+4	2.26e+4	3.54e+4	6.84e+4	1.32e+5	2.48e+5	3.44e+5	1.15e+5	1.69e+5
MDN50k	6.43e+3	7.84e+3	1.35e+4	2.67e+4	5.76e+4	1.11e+5	1.64e+5	4.88e+4	6.58e+4
MDN100k	4.35e+3	5.12e+3	8.28e+3	1.87e+4	4.20e+4	8.80e+4	1.32e+5	3.65e+4	5.32e+4
Autofit	2.62e+3	2.77e+3	3.07e+3	3.62e+3	4.71e+3	7.38e+3	1.25e+4	7.23e+3	7.76e+4
$\chi ^2_{\nu =2200}$	2.09e+3	2.12e+3	2.16e+3	2.2+3	2.24e+3	2.29e+3	2.31e+3	2.20e+3	6.60e+1

**Table 5:** The average coverage score (ACS) and the median relative error (MRE) values for test data from datasets A, B, and C for two selected EDP parameters that exhibit the highest and lowest point estimate accuracy.

Metric	Test Data A		Test Data B		Test Data C	
	W in Layer 1	D in Layer 1	Ni in Layer 1	F in Layer 3	La in Layer 1	H in Layer 1
ACS	0.330	0.272	0.251	0.208	0.261	0.162
MRE	0.0046	0.0087	0.0095	0.0306	0.0127	0.4016

Looking at Table 2, as expected, using more training data improves the $\chi ^2$ values. However, even with 100,000 training data, our model cannot completely replicate the ideal $\chi ^2$ distribution. In fact, using the previously mentioned goodness-of-fit test, we can say that $\lesssim 75\%$ of the predicted EDPs from MDN50k are accepted. Now, looking at the $\chi ^2$ from the Autofit, we can see that it aligns well with the $\chi ^2_{\nu =700}$ until roughly the 50th percentile. The $\chi ^2$ values are also better than that from MDN. However, looking at the average and the spread, the $\chi ^2$ values from the Autofit are much worse than even MDN10k. This comparison highlights the strengths and weaknesses of both methods: the Autofit, if it properly converges to a global minimum, performs exceptionally well. However, if convergence fails, the fitted EDPs may diverge significantly from the true values. In contrast, while our deep learning model has slightly worse performance than the *converged* automatic fit method, its performance is relatively consistent across all the test data.

Turning our focus to dataset B (with an RoI length of 1310), whose $\chi ^2$ profile is reported in Table 3, we observe that the $\chi ^2$ values from MDN50k and MDN100k are very good. Employing the same goodness-of-fit test as before, we find that around 75% of the predictions from these models are accepted. Comparing the results from Autofit and the MDNs, we see that Autofit performs better, with 90% of its predictions passing the test. However, the mean and spread of the $\chi ^2$ values for Autofit predictions are much larger than those for the MDNs, indicating extreme outliers in less than 5% of the Autofit predictions.

Looking at Table 4 (with an RoI length of 2200), which displays the $\chi ^2$ profile from dataset C, we observe significantly higher $\chi ^2$ values than expected for both the MDN and Autofit methods. Even at lower $\chi ^2$ percentiles, the predictions from these methods fail to meet the goodness-of-fit criteria. This underscores the challenges faced by both methods in inferring elemental depth profiles characterized by high complexity and ambiguity.

We note here that our Autofit implementation uses the Nelder–Mead minimizer, which is known to be inefficient in high-dimensional spaces^20^. The failure of the Autofit method for dataset C may be attributed to the limitation of the *Maxfev*=$200\times D$ stopping condition in the Nelder–Mead algorithm. In other words, a substantially higher number of $\chi ^2$ function calls are necessary, which would consequently require longer computation times.

From Table 4, it is evident that the $\chi ^2$ values obtained from the Autofit method are consistently better than those from our ML models. Dataset C thus exemplifies a case where the Autofit approach shines: whenever the degrees of ambiguity and complexity of the samples are high enough, directly minimizing the $\chi ^2$ function, as in the Autofit approach, is a more effective method for achieving the lowest $\chi ^2$ values compared to simply guessing the global minimum of the $\chi ^2$ function from the training data. This finding also serves as a cautionary note when interpreting previous machine learning studies^8–11^ on RBS spectra, which have shown promising results for machine learning approach but typically involve relatively simple sample structures (with fewer than 11 EDP parameters). Our study demonstrates that in cases of high complexity (measured in terms of the number of EDP parameters) and ambiguity (in terms of cross-section overlaps), conventional techniques such as Autofit, despite their slower nature, emerge as the superior option.

Our discussion here highlights the distinct performance characteristics of the Autofit and MDN models in reproducing ideal $\chi ^2$ distributions. For a relatively simple to intermediate sample structures, while the Autofit excels under optimal convergence, the MDN models demonstrate more uniform performance across all the test data. For the more complicated case represented by dataset C, Autofit outperforms the MDN models. However, challenges still persist for both approaches in achieving optimal $\chi ^2$ values.

### Uncertainty of predictions

In the introduction, we highlighted our primary motivation for opting for a probabilistic model over a simple regression model in IBA analysis: the ability to estimate the uncertainty of model predictions. As discussed in section “Methods”, given a posterior density $p({\textbf {y}}|x)$, the uncertainty $\Delta y_i$ associated with a point prediction $\hat{y}_i = \langle y_i \rangle$ is computed as the standard deviation derived from the posterior, expressed as $\Delta y_i = \sqrt{\langle (y_i-\hat{y}_i)^2\rangle }$. In this section, we discuss the accuracy of these uncertainty estimates.

**Figure 8:**
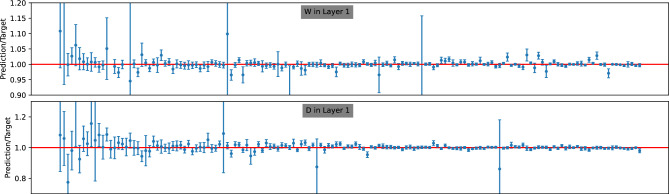
Prediction-to-target ratio for the 150 samples randomly chosen from the test data from dataset A. Following selection, these 150 samples are arranged in ascending order.

**Figure 9:**
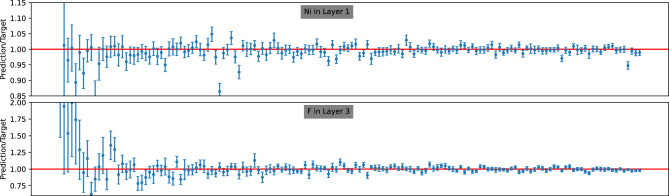
Prediction-to-target ratio for the 150 samples randomly chosen from the test data from dataset B. Following selection, these 150 samples are arranged in ascending order.

**Figure 10:**
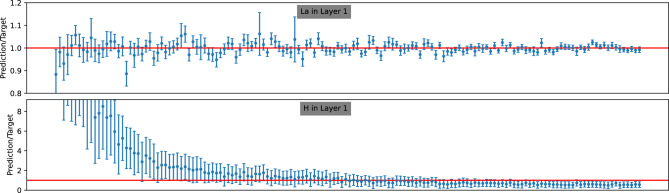
Prediction-to-target ratio for the 150 samples randomly chosen from the test data from dataset C. Following selection, these 150 samples are arranged in ascending order.

Several techniques exist to gauge the accuracy of uncertainty estimates in reflecting true uncertainty. The concept of uncertainty calibration, as discussed in the literature (see for example^32,33^) is one such technique. In this work, however, we adopt more pragmatic approach by directly comparing the uncertainty prediction to the predictor systematic error, which is defined as the distance between the prediction and the target values. This approach provides a straightforward and intuitive means of assessing the reliability of our uncertainty estimates.

To assess how close is the uncertainty estimate to the predictor systematic error, we introduce the concept of average coverage score (ACS) $\Gamma _i$ for the *i*-th EDP parameter as:

17 $$\begin{aligned} \Gamma _i&= \frac{1}{N_{\text {test}}} \sum _k^{N_{test}} \gamma _i^{(k)}, \qquad \qquad -1\le \Gamma _i \le 1 \end{aligned}$$

Here, the coverage score $\gamma _i^{(k)}$ for the *k*-th data is defined as (the index *k* is suppressed)

18 $$\begin{aligned} \gamma _i = \frac{\Delta y_i- \Delta y_{i, 0}}{\Delta y_i+ \Delta y_{i, 0}}, \qquad \qquad -1 \le \gamma _i \le 1 \end{aligned}$$

where $N_{test}$ is the number of the test data, $\Delta y_i$ represents the model’s uncertainty estimate for the *i*-th EDP parameter, and $\Delta y_{i, 0}$ is the predictor systematic error, defined as:

19 $$\begin{aligned} \Delta y_{i, 0} = | \hat{y}_{i} - y_{i, 0}| \end{aligned}$$

where $y_{i, 0}$ denotes the target value for the EDP parameter $y_i$.

Given an ACS value $\Gamma _i$, a negative ACS indicates an overall underestimation of predictor systematic errors, while a positive ACS suggests the opposite. This can be understood by looking at the extreme points of the individual coverage score. For instance, the value $\gamma _i \approx -1$ represents an severe under-coverage (with true/target value lies outside of the the estimated error bars) such that $\Delta y_i\ll \Delta y_{i, 0}$. Conversely, $\gamma _i \approx 1$ represents severe over-coverage, as now $\Delta y_i\gg \Delta y_{i, 0}$.

While the ACS isn’t directly related to uncertainty calibration, it’s intriguing to determine the range of ACS values that would indicate a perfectly calibrated uncertainty. Establishing this range should provide clarity on what ACS values are considered “reasonable”. We note that an uncertainty estimate $\sigma _p$ in the form of credibility interval at quantile $p\in [0,1]$ is said to be calibrated if the fraction of the number of instance of the interval covering the true values match the predicted probability, which is *p*, see^32^. Now, let’s suppose that the uncertainty estimate $\Delta y_i$ is associated with the *q* quantile of the distribution. In the case of a Gaussian posterior, the uncertainty estimate, defined by the variance of the distribution (as in our case), corresponds to $q=0.68$. If the uncertainty is perfectly calibrated at quantile *q*, there are a fraction *q* of over-coverage cases, or equivalently, $1-q$ under-coverage cases. Given that an under-coverage case is represented by $\gamma _i\in [-1,0]$ and an over-coverage case is represented by $\gamma _i \in [0,1]$, the ACS value must satisfy:

20 $$\begin{aligned} q-1 \le \Gamma _i \le q \qquad \qquad \text {(If }\Delta y_i \text {is calibrated at quantile q)} \end{aligned}$$

Given $q=0.68$ in our case, the range [-0.32, 0.68] thus defines a range of ACS values that we consider reasonable.

In Table 5, we show the ACS values and median relative error (MRE) for the test data from dataset A, B and C, focusing on two EDP parameters: one that corresponds to the best point-estimate accuracy (in terms of MRE) and the other exhibiting the worst accuracy. Note that we use our default model, MDN50k, from each dataset to generate both point and error bar predictions.

It is evident that the ACSs is positive for all test data across the three datasets, indicating an overall tendency for the uncertainty estimates to overestimate the predictor systematic error. Interestingly, we observe an inverse relationship between the ACS and the MRE. This observation hints at the possibility that the reduced ACS value may stem from the increased magnitude of $\Delta y_{i, 0}$ due to less accurate point estimates of the EDP parameters. Despite observing some variation in ACS values across the three datasets, they consistently fall within an acceptable range in the sense of uncertainty calibration.

To visualize how well the error bars from the uncertainty estimates to encompass the target EDP parameter value from the test data, we define the prediction-to-target ratio and its uncertainty as follows:

21 $$\begin{aligned} R_i = \frac{\hat{y}_{i}}{y_{i, 0}}, \qquad \Delta R_i = \frac{1}{y_{i, 0}} \Delta y_{i} \end{aligned}$$

Here, as before, $\hat{y}_{i}$ and $y_{i, 0}$ represent the *i*-th predicted and target EDP parameters, respectively, while $\Delta y_{i}$ denotes the uncertainty prediction for $y_{i}$. By plotting $R_i$ instead of $y_i$, it is easy to compare the uncertainty across different points, as now the perfect point prediction corresponds to $R_i=1$ for all values of EDP parameters.

In Figs. 8, 9, and 10, we present the ratio *R* for 150 randomly selected samples from the test data of datasets A, B, and C, respectively. Each figure includes an upper panel displaying the *R* ratio for the parameter with the best predicted values (i.e., the lowest MRE), while the bottom panel illustrates parameters corresponding to the largest MRE. The target values in all figures are sorted in ascending order.

Upon closer inspection of these figures, a discernible trend emerges: the size of the error bars generally decreases as the target values increase. This observation suggests that achieving accurate predictions for parameters with larger values is relatively more feasible compared to smaller ones. Furthermore, across all three datasets, the error bars predominantly envelop the ideal value ($R=1$), indicating an overall tendency for overestimation. However, this tendency is less pronounced for the $H_1$ parameter in dataset C. This observation thus aligns well with their respective ACS values.

In conclusion, our analysis reveals insightful patterns regarding the accuracy and consistency of uncertainty estimates across different datasets. Despite minor overestimations observed, the overall performance demonstrates the capability to encapsulate target EDP parameter values within the predicted error bars.

### Combining deep learning and automatic fitting method

Our experiments with three datasets of varying complexities demonstrated that while our model performs well on datasets A and B, it still struggles to replicate the ideal $\chi ^2$ distribution for the test data in dataset C. Observing that an automatic fitting method performs remarkably well when it converges to a global minimum suggests that a combined approach using MDN and automatic fitting-where the initial point of the automatic fit comes from the MDN’s prediction-should theoretically lead to significantly improved model performance. In the context of automatic fitting, it also offers added advantage of reducing user intervention in providing an educated guess of the initial parameters.

**Table 6:** Statistical profile (percentiles, mean and standard deviation) of $\chi ^2$ of the test data of dataset A, B and C, obtained using MDN50k+Autofit method.

Method	Percentiles							Mean	Std
	5th	10th	25th	50th	75th	90th	95th		
$\chi ^2_{\nu =700}$	640	652	674	699	725	748	763	700	37
Dataset A	619	630	652	678	705	733	753	695	187
$\chi ^2_{\nu =1310}$	1227	1245	1275	1309	1344	1376	1395	1310	51
Dataset B	988	1035	1111	1190	1272	1328	1361	1187	115
$\chi ^2_{\nu =2200}$	2092	2115	2155	2199	2244	2285	2310	2200	66
Dataset C	2260	2311	2433	2627	2987	3758	4880	3051	2015

We acknowledge that a similar combined approach was previously employed in^34^, albeit without a comparative analysis against the standard Autofit or machine learning methods. In this section, we will focus on comparing the performance of the individual MDN and Autofit methods with the combined approach.

In Table 6, we present the statistical profiles of $\chi ^2$ for 2000 test data points from datasets A, B, and C. For these datasets, the EDP parameters were predicted using a combined MDN+Autofit method. The MDN was trained on 50,000 training data samples for each dataset, while the Autofits were performed with a quarter of the *Maxfev* used in our standard Autofit method. This means that *Maxfev* is set to $50\times D$, where *D* is the number of EDP parameters. The rationale behind reducing the *Maxfev* values is that the ML approach should already provide a good starting point for the Autofits, thus warranting a decrease in *Maxfev*.

Upon analyzing Table 6, it is evident that the resulting $\chi ^2$ values are lower than those obtained using both the Autofit and MDN methods, indicating that the combined method outperforms both. Regarding the goodness-of-fit (GoF) test, we observe that for datasets A and B, over 95% of predictions satisfy the criteria, marking a substantial enhancement over the individual methods where only around 75% of test cases are deemed acceptable. In dataset C, now 10% of test cases fulfill the GoF criteria. This is an improvement, as nearly all predictions for the test data fail to meet the criteria before.

Analyzing the runtime of this combined method in Table 1, we find that it requires less computing time than the standard Autofit method. For datasets B and C, the runtimes are roughly one third of those from the standard Autofit method. This underscores the superiority of this method in terms of both accuracy and efficiency.

## Conclusions

In this study, we utilized a mixture density network (MDN) architecture, consisting of a convolutional neural network (CNN), a gated recurrent unit (GRU) as the encoder network (EN), and a multilayer perceptron (MLP) as the mixture density head, to predict the posterior distribution of elemental depth profile parameters (EDPs) from input spectra. Training was conducted on three datasets of varying complexities: dataset A (4 EDP parameters), dataset B (15 EDP parameters), and dataset C (30 EDP parameters). While our machine learning (ML) approach generally yielded similar accuracy metrics to the standard Autofit method for datasets A and B, Autofit significantly outperformed the MDN model for dataset C, highlighting the need for caution when applying ML approaches to complex sample structures. Additionally, we explored a combined ML+Autofit approach, which yielded significant enhancements in the $\chi ^2$ distribution of test data compared to both approaches while requiring fewer computational resources than Autofit.

Given that this work utilizes simulated spectra, it is anticipated that the accuracy of predictions may decline when applied to real experimental spectra. However, previous studies have demonstrated that using simulated spectra for training data can yield promising results when extrapolated to experimental datasets, suggesting that this trend may hold true for our study as well. It is also important to acknowledge that our method relies on SIMNRA to generate training data, which depends on fundamental databases such as cross-section and stopping power. Consequently, any predictions made by our machine learning model will inherently reflect the uncertainties present in these fundamental databases. Despite these challenges, the success of previous studies using similar methodologies and the robustness of fundamental databases suggest that our approach holds promise for real-world applications, provided appropriate considerations and validations are undertaken.

### Supplementary Information

Supplementary Information.

## Data Availability

The datasets used and/or analysed during the current study available from the corresponding author on reasonable request.
