# Supplementary material for: Analysis of Rutherford backscattering spectra with CNN-GRU mixture density network
Source: Sci Rep. 2024 Jul 23;14:16983. doi: 10.1038/s41598-024-67629-y (PMC11266510; doi:10.1038/s41598-024-67629-y)
Supplement: Supplementary file 1 — Supplementary Information. [file 41598_2024_67629_MOESM1_ESM.pdf]

# Analysis of Rutherford Backscattering Spectra with CNN-GRU Mixture Density Network : Supplementary Material

**Khoirul Faiq Muzakka[1,*], Sören Möller[1], Stefan Kesselheim[2], Jan Ebert[2], Alina Bazarova[2], Helene Hoffmann[3], Sebastian Starke[3], and Martin Finsterbusch[1]**

[1]Institut für Energie- und Klimaforschung, Forschungszentrum Jülich GmbH, 52428 Jülich, Germany
[2]Jülich Supercomputing Centre, Forschungszentrum Jülich GmbH, 52428 Jülich, Germany
[3]Helmholtz-Zentrum Dresden-Rossendorf, 01328 Dresden, Germany
[*]Email : k.muzakka@fz-juelich.de

## Supplementary Material A: Ablation Study Results with Dataset A

In this section, we present the outcomes of our ablation study, aimed at evaluating the influence of various components and configurations of our model on its predictive efficacy on dataset A with a training data size of 10000. This also functions as an overview of the machine learning (ML) models we have explored thus far. It's worth noting that we did not conduct extensive hyperparameter tuning; instead, we manually explored several configurations and selected the most optimal one. In table S1, we present the model setup and hyperparameters of both MDN and point estimator models that we experimented with. Additionally, in Table S2, we display the $\chi^2$ profiles generated by each model.

Upon examining the $\chi^2$ profiles from the point-estimator models in Table S2, we observed that neural-network-based (NN-based) methods generally outperform non-neural network models such as XGBoost and Random Forest. Moreover, among the NN-based approaches, the hybrid method CNN+GRU demonstrates the highest performance. Shifting focus to MDN models, it is evident that those incorporating CNN as the encoder network (EN) outperform point-estimator models using only CNN. This trend is consistent for MLP, GRU, and the combined CNN+GRU as well. Ultimately, the MDN model with the combined CNN+GRU as EN emerges as the top performer among all models considered in this study.

## Supplementary Material B: MDN Training

Training mixture density network is notorious for being prone to numerical instability problem. In this section, we delve into our strategies for mitigating this issue.

As usual, training is done by minimizing a loss function. In our case, the loss function is the negative log posterior. Given $N_{train}$ training data $\{(x^{(k)},\mathbf{y}^{(k)}),\ k=1,2,...,N_{train}\}$, the joint posterior distribution is given by $p(\mathbf{y}^{(1)},...,\mathbf{y}^{(N_{train})}|x^{(1)},...,x^{(N_{train})}) = \prod_k p(\mathbf{y}^{(k)}|x^{(k)})$. Given Eqs. (7) in the main article, the loss function $\mathscr{L}$ is therefore given by

$$\mathscr{L}(\theta) = -\frac{2}{N_{train}} \log p(\mathbf{y}^{(1)},...,\mathbf{y}^{(N_{train})}|x^{(1)},...,x^{(N_{train})}) = -\frac{2}{N_{train}} \sum_k^{N_{train}} \log\left[\sum_i^M \exp\left(-\frac{\phi_i(x^{(k)},\mathbf{y}^{(k)})}{2}\right)\right] + const \tag{a}$$

Note that a prefactor $2/N_{\text{train}}$ is added in order for the loss function to reduce to mean-squared-error (MSE) loss when $M=1$ and the Gaussian posterior has a constant (homoscedastic) variance.

Given the loss function (a), the source of the numerical problems often encountered during the training of Mixture Density Networks (MDN), particularly with large training datasets ( > 20,000 training samples), can be identified as follows:

1. Underflow/overflow from the exponent in (a). To mitigate this problem, we use logsumexp-trick:

$$\log\left(\sum_k e^{\lambda_k}\right) = \lambda_{k_0} + \log\left(1 + \sum_{k\neq k_0} e^{\lambda_k - \lambda_{k_0}}\right) \tag{b}$$

   where $k_0 = \arg\max_k \lambda_k$. We note here that even after using this trick, overflow/underflow can still occur when $|\lambda_k - \lambda_{k_0}|$ is large. This typically happens in the initial stage of training, where the optimizer tried to wildly explore the parameter space.

| Model | Setup and Hyperparameters |
|---|---|
| | MDN Model with different EN Modules |
| MDN CNN+GRU | EN CNN : N_Kernel_per_layer : [45, 35, 30, 25], Kernel_size_per_layer : [7, 5, 5, 3], Stride_per_layer : [1, 2, 1,2 ], No pooling. EN GRU : N_hidden_states=128, 2 layers, bidirectional, dropout=0.2. MDH : no hidden node for $\mu$ FF, 300 hidden nodes for $\sigma$ FF, no hidden nodes for $\pi$ FF. M=1. |
| MDN CNN | EN CNN : N_Kernel_per_layer : [20, 30, 40, 50, 60], Kernel_size_per_layer : [7, 5, 5, 3, 3], Stride_per_layer : [1, 2, 1,2, 1 ], No pooling. EN GRU : 2 layers, bidirectional, dropout=0.2. MDH : no hidden layer for $\mu$ FF, 300 hidden nodes for $\sigma$ FF, no hidden nodes for $\pi$ FF. M=1. |
| MDN GRU | EN GRU : N_hidden_states=128, 2 layers, bidirectional, dropout=0.2. MDH : no hidden node for $\mu$ FF, 300 hidden nodes for $\sigma$ FF, no hidden nodes for $\pi$ FF. M=1. |
| MDN MLP | EN MLP : Hidden nodes = [150, 100], Spectrum length after EN : 50. MDH : no hidden node for $\mu$ FF, 300 hidden nodes for $\sigma$ FF, no hidden nodes for $\pi$ FF. M=1. |
| | Point Estimator Models |
| XGBoost | max_depth=12, learning_rate=0.2, reg_lambda=2.0, other parameters are not changed from the default values as implemented in the XGboost package[**?** ]. |
| Random Forest | max_depth 0 20, n_estimators=64, other parameters are not changed from the default values as implemented in the Scikit-learn package[**?** ]. |
| MLP | Hidden nodes : [400, 200], Leaky-relu non-linearity. |
| CNN | N_Kernel_per_layer : [20, 30, 40, 50, 60], Kernel_size_per_layer : [7, 5, 5, 3, 3], Stride_per_layer : [1, 2, 1,2, 1 ], No pooling. |
| LSTM | N_hidden_states=128, 2 layers, bidirectional, dropout=0.2. |
| GRU | N_hidden_states=128, 2 layers, bidirectional, dropout=0.2. |
| CNN+GRU | CNN : N_Kernel_per_layer : [45, 35, 30, 25], Kernel_size_per_layer : [7, 5, 5, 3], Stride_per_layer : [1, 2, 1,2 ], No pooling. GRU : N_hidden_states=128, 2 layers, bidirectional, dropout=0.2. |

**Table S1.** Model setup and hyperparameters of the machine learning models investigated for dataset A. Key notations include: EN (encoder network), MDH (mixture density head), XGBoost (extreme gradient boosting), MLP (multi-layer perceptron), CNN (convolutional neural network), GRU (gated recurrent unit network), LSTM (long short-term memory network), N_Kernel_per_layer (number of convolutional kernels per layer), Stride_per_layer (stride per convolutional layer), N_hidden_states (number of hidden states in RNN), FF (feed forward unit), M (number of Gaussian mixtures), and $\mu$, $\sigma$, $\pi$ (mean, standard deviation, and mixture coefficient of the MDN). Additionally, MDN CNN (GRU, MLP, CNN+GRU) represents MDN with CNN (GRU, MLP, CNN+GRU combined) as the Encoder (EN) module. The MDN CNN+GRU model serves as the final model used throughout this study.

2. When $\sigma$ and $\pi$ is too small, instability in the calculation of $\phi$ occurs. The instability manifests in the occurence of *-inf* or *inf*, which further cause *NaN* gradients. To avoid the instability, we rewrite the second term in Eqs. (8) of the main article as

$$\log\left(\frac{\pi_i(x)}{\prod_j^D \sigma_j^i(x)}\right) = \log \pi_i - \sum_j^D \log(\sigma_j^i) \tag{c}$$

and limit the values of $\sigma$ and $\pi$. To allow smooth derivatives at the boundary, this restriction is implemented as adding a small value $\varepsilon$ to the original value of $\sigma$ and $\pi$. For $\sigma$, we set $\varepsilon = 1\times 10^{-10}$, while for $\pi$, we set $\varepsilon = 1\times 10^{-20}$.

As mentioned before, the logsumexp-trick does not entirely solve the numerical instability. Therefore, we propose a new loss function $\mathscr{L}'$ which is semi-equivalent, in the following sense, to the original loss $\mathscr{L}$. The new loss is defined as

$$\mathscr{L}'(\theta) = \frac{1}{N_{train}}\frac{1}{M}\sum_k^{Ntrain}\sum_i^M \phi_i(x^{(k)}, \mathbf{y}^{(k)}) \tag{d}$$

Compared to the previous loss function (a), we can see there is no longer log or exponent functions appearing, and therefore should be more numerically stable. As it contains fewer non-linearities, it is also easier to minimize. Now we give the following proposition:

| Method | Percentiles | | | | | | | Mean | Std |
|---|---|---|---|---|---|---|---|---|---|
| | 5th | 10th | 25th | 50th | 75th | 90th | 95th | | |
| $\chi^2_{v=700}$ | 640 | 652 | 674 | 699 | 725 | 748 | 763 | 700 | 37 |
| Autofit | 626 | 640 | 667 | 707 | 1360 | 6083 | 15380 | 5640 | 28309 |
| MDN Model with different number $M$ of Gaussian mixtures | | | | | | | | | |
| MDN CNN+GRU (M=1) | 667 | 686 | 725 | 801 | 1066 | 2722 | 3972 | 1308 | 1720 |
| MDN CNN+GRU (M=5) | 670 | 689 | 732 | 817 | 1080 | 2470 | 3831 | 1264 | 1430 |
| MDN Model with different EN modules | | | | | | | | | |
| MDN CNN | 732 | 772 | 917 | 1420 | 3085 | 5561 | 8241 | 2851 | 6495 |
| MDN GRU | 701 | 735 | 837 | 1138 | 2136 | 4275 | 5246 | 2016 | 2615 |
| MDN MLP | 712 | 750 | 872 | 1405 | 3621 | 7220 | 13551 | 4069 | 11046 |
| Point Estimator Models | | | | | | | | | |
| XGBoost | 956 | 1172 | 1929 | 4131 | 8061 | 17724 | 28559 | 9864 | 33622 |
| Random Forest | 868 | 998 | 1449 | 2996 | 5953 | 19014 | 44995 | 12133 | 42720 |
| MLP | 752 | 815 | 1081 | 1880 | 4179 | 9749 | 22592 | 7294 | 46329 |
| CNN | 761 | 817 | 1000 | 1551 | 3328 | 5542 | 9076 | 2967 | 4866 |
| LSTM | 801 | 898 | 1270 | 2377 | 4404 | 7718 | 13778 | 5550 | 33074 |
| GRU | 751 | 819 | 1035 | 1732 | 3531 | 6894 | 12475 | 3869 | 8504 |
| CNN+GRU | 725 | 769 | 877 | 1201 | 2435 | 4487 | 6649 | 2465 | 4969 |

**Table S2.** Statistical profile (percentiles, mean and standard deviation) of $\chi^2$ of the test data of dataset A calculated using various ML models with a training data size of 10 000.

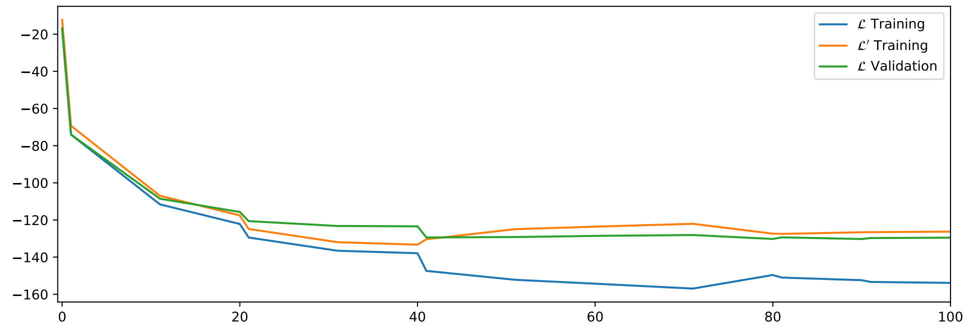


**Figure S1.** Learning curve of MDN training on dataset C with 50 000 training data.

**Proposition 1.** *For any given network parameters $\theta$,*

$$\mathscr{L}(\theta) \leq -2\log M + \mathscr{L}'(\theta) < \mathscr{L}'(\theta) \tag{e}$$

*and the equality occurs when $\phi_1 = \phi_2 = ... = \phi_M$.*

*Proof.* The inequality (e) is a direct consequence of the Jensen inequality, that for any concave function $\varphi$ (such as log),

$$\varphi\left(\sum_j \alpha_j x_j\right) \geq \sum_j \alpha_j \varphi(x_j), \qquad \sum_j \alpha_j = 1 \tag{f}$$

For conciseness, let's denote $\phi_i^k = \phi_i(x^{(k)}, \mathbf{y}^{(k)})$. Then from (a) and (f),

$$\mathscr{L}(\theta) = -\frac{2}{N_{train}} \sum_k \log\left[\sum_i \exp\left(-\frac{\phi_i^k}{2}\right)\right] = -\frac{2}{N_{train}} \sum_k \log\left[\sum_i \frac{1}{M} \exp\left(-\frac{\phi_i^k}{2} + \log M\right)\right]$$
$$\leq -\frac{2}{N_{train}} \sum_k \sum_i \frac{1}{M}\left(\log M - \frac{\phi_i^k}{2}\right) = -2\log M + \mathscr{L}'(\theta) < \mathscr{L}'(\theta)$$

□

Given that $\mathscr{L} < \mathscr{L}'$, minimizing $\mathscr{L}'$ also minimize the loss $\mathscr{L}$. However, as $\mathscr{L}'$ only serves as an upper bound to the $\mathscr{L}$, the minimum of $\mathscr{L}'$ is therefore not necessarily the minimum of $\mathscr{L}$. Thus, further refinement is necessary. In practice, our training strategy for the Mixture Density Network (MDN) involves initially training with the $\mathscr{L}'$ loss function and subsequently refining the optimization by using $\mathscr{L}$. Specifically, we use $\mathscr{L}'$ loss for the first 40% of the total epochs and then transition to using the $\mathscr{L}$ loss for the remaining 60% of the total epochs.

In Figure S1, we show the learning curve of our MDN model, trained on a dataset C with 50 000 training instances over 100 epochs. The MDN uses 10 Gaussian components. Despite the high number of Gaussian components and output parameters, no numerical instabilities are encountered during training. Observing Figure S1, it is evident that $\mathscr{L}'$ consistently remains larger than $\mathscr{L}$, validating the proposition. We can also observe that from epoch 40 onwards, $\mathscr{L}$ diverges from $\mathscr{L}'$. This divergence occurs because training with the $\mathscr{L}$ loss begins at epoch 40, further refining the true loss $\mathscr{L}$ while still maintaining $\mathscr{L} < \mathscr{L}'$.
